# A Pilot Study of Rare Renal Amyloidosis Based on FFPE Proteomics

**DOI:** 10.3390/molecules26237234

**Published:** 2021-11-29

**Authors:** Shuang Meng, Wenwen Xia, Li Xia, Li Zhou, Jing Xu, Xiaoxia Pan, Liyuan Meng

**Affiliations:** 1 Department of Core Facility of Basic Medical Sciences, Shanghai Jiao Tong University School of Basic Medicine, Shanghai 200025, China; mengshuang92@shsmu.edu.cn (S.M.); lixia@shsmu.edu.cn (L.X.); zhouli_bit@shsmu.edu.cn (L.Z.); 2 Department of Pathology, Ninth People’s Hospital, Shanghai Jiao Tong University School of Medicine, Shanghai 200011, China; xiawenwenliuyedao@163.com; 3 Department of Nephrology, Ruijin Hospital, Shanghai Jiao Tong University School of Medicine, Shanghai 200025, China; xj11564@rjh.com.cn

**Keywords:** proteomics, FFPE, renal amyloidosis, immunoglobulin light chain amyloidosis (AL), lysozyme amyloidosis (ALys)

## Abstract

Renal amyloidosis typically manifests albuminuria, nephrotic-range proteinuria, and ultimately progresses to end-stage renal failure if diagnosed late. Different types of renal amyloidosis have completely different treatments and outcomes. Therefore, amyloidosis typing is essential for disease prognosis, genetic counseling and treatment. Thirty-six distinct proteins currently known to cause amyloidosis that have been described as amyloidogenic precursors, immunohistochemistry (IHC) or immunofluorescence (IF), can be challenging for amyloidosis typing especially in rare or hereditary amyloidosis in clinical practice. We made a pilot study that optimized the proteomics pre-processing procedures for trace renal amyloidosis formalin-fixed paraffin-embedded (FFPE) tissue samples, combined with statistical and bioinformatics analysis to screen out the amyloidosis-related proteins to accurately type or subtype renal amyloidosis in order to achieve individual treatment. A sensitive, specific and reliable FFPE-based proteomics analysis for trace sample manipulation was developed for amyloidosis typing. Our results not only underlined the great promise of traditional proteomics and bioinformatics analysis using FFPE tissues for amyloidosis typing, but also proved that retrospective diagnosis and analysis of previous cases laid a solid foundation for personalized treatment.

## 1. Introduction

Amyloidosis is a condition characterized by deposition of autologous proteins as extracellular fiber aggregates. This class of diseases is heterogeneous and continuous expansion. So far, 36 distinct precursor proteins are known to cause amyloidosis in humans [1]. Amyloidosis can cause systemic or local lesions; kidney was the most commonly affected organ by systemic amyloidosis [2,3,4]. Renal amyloidosis is a rare and intractable protein misfolding disorder which prompts progressive renal insufficiency [5]. The common forms of systemic amyloidosis include immunoglobulin light chain amyloidosis (AL), amyloid A amyloidosis (AA) and leukocyte chemotactic factor 2 (LECT2) amyloidosis. Hereditary or familial amyloidosis is another group including fibrinogen Aα chain, transthyretin (TTR), apolipoprotein A-I and apolipoprotein A-II, lysozyme, gelsolin and cystatin C [6,7]. Renal amyloidosis cases are predominantly AL and ALECT2 amyloidosis, but sometimes rare hereditary or familial amyloidosis are also found to be involved in the renal pathogenesis [8,9,10]. Immunoglobulin (Ig) light chain (LC) amyloidosis (AL) is characterized by an abnormal extracellular deposition of a monoclonal Ig LC protein of either lambda (λ) or kappa (κ) isotype [11]. Most forms of amyloidosis are progressive and fatal [12]; different types have similar manifestations but completely different treatment strategies. Early diagnosis and accurate amyloid typing is a key diagnostic step for better understanding of their pathogenesis for rational and often successful treatment strategies [13,14].

Renal pathology is the main basis for clinical diagnosis of renal amyloidosis. Apple green birefringence of the deposits stained with Congo red and viewed in polarized light is still the gold standard for histological. Clinical pathological classification is mainly based on antibody-based immunohistochemical (IHC) or Immunofluorescence (IF) staining technologies, which determine the type of disease through the type of fibrin identified [7,12,15,16]. AL amyloid fibers derive from truncated immunoglobulin light chain; most variable regions are difficult to detect by commercial antibodies (mainly constant regions). There are no commercial antibodies for some hereditary and rare cases. Standard panels are typically designed to detect only three amyloid types (AL, ATTR and AA); hence, it is more difficult to identify rare amyloid types [4]. When the amount of amyloid deposited in the tissue is small or the distribution of amyloid protein in the paraffin is uneven, it can lead to missed diagnosis or difficult typing [17]. The diagnosis or early renal amyloidosis is occasionally neglected by depending only on light micrograph and IF.

Proteomics aims to identify all protein information in samples such as cells or tissues. With continuously developing of high-resolution mass spectrometry technology, proteomics has become a powerful tool for the identification of fibroid protein. It is applied to the diagnosis of renal amyloidosis and can efficiently and unbiasedly identify all the amyloid precursor protein information in a diseased tissue, without requiring a priori knowledge of the candidate amyloid protein [18,19]. At present, MS-based proteomics has been used clinically to assist diagnosing amyloidosis and it has become one of the gold standards for amyloidosis typing [20]. The criteria for proteomics aiding diagnosis amyloidosis in clinical practice: all the proteins identified with the highest abundance (or the number of spectrums) were determined to be corresponding amyloid fibril protein. Different amyloidosis types were determined according to the type of amyloid fibril protein [1,21]. SAP and apolipoprotein E are universally associated with all types of amyloid fibrils, and these components were known as ‘amyloid signatures’ [8]. FFPE-based proteomics is unbiased to identify all amyloid fiber protein types in a single assay, unlike immunologic-based techniques that a single test would only identify a single amyloid fibril protein type.

The diagnosing and typing method by proteomics developed by Ahmed Dogan’s research team has been used for clinical diagnosis of amyloidosis. The National Amyloidosis Center of the United Kingdom has also carried out proteomics to classify amyloidosis. This method can be used for fresh or FFPE tissue [4,21,22]. FFPE archives a biobank and invaluable resource for clinical diagnosis and biomarker research; however, the clinical pathological FFPE tissue section contains very little tissue. After formaldehyde-fixed paraffin embedding, the proteins exist in a cross-linked state. Proteomics research has good sensitivity and specificity even if it uses trace FFPE tissue samples, and does not require antibodies in diagnosing amyloidosis. It is usually difficult to diagnose on routine IF and IHC methods [18,22]. Proteomics has revolutionized the field of amyloid diagnosis, which is considered to be the new gold standard approach for disease typing. Proteomics now has a profound impact in the clinical management of amyloid diseases [23]. However, until now, there is not a traditional FFPE-based proteomics and bioinformatics analysis used for the subtype of renal amyloidosis.

## 2. Results

### 2.1. FFPE-Based Proteomics Discriminate Renal Amyloidosis and Control Patient

**Figure 1:**
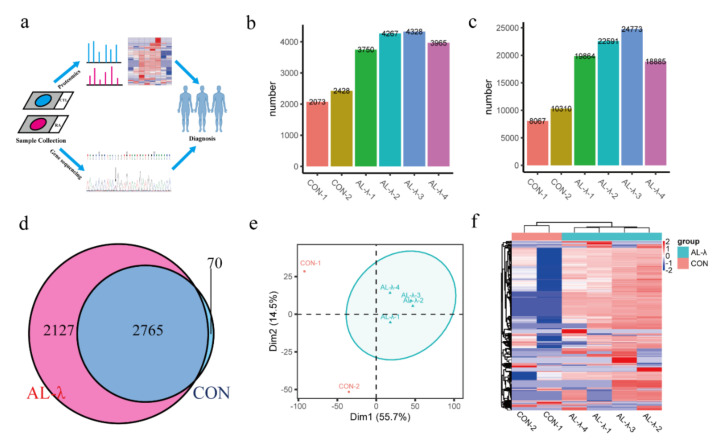
(**a**). FFPE-based proteomics and bioinformatics analysis workflow. (**b**). Bar chart of total number of identified protein groups in control and AL-λ patients. (**c**). Bar chart of total number of identified peptides in control and AL-λ patients. (**d**). Venn analysis of protein groups in control and AL-λ patients. (**e**). Principal component analysis (PCA) of control and AL-λ patients based on proteomics data; ellipses indicate AL patients. (**f**). Heat map of control and AL-λ patients with all identified proteins; control patients are separated form AL-λ patients.

Renal amyloidosis is a rare disease and the biopsy is rare in clinical application. FFPE tissues are collected from glass slides, followed by extracting protein and de-crosslinking for LC-MS/MS analysis and bioinformatics analysis. There were seven patients in this study including two minor glomerular disease patients as control group, four AL-λ patients and one rare lysozyme amyloidosis (Alys) patient. The experimental workflow is as displayed in Figure 1a.

Numbers of identified proteins and peptides are shown in Figure 1 (Figure 1b,c). There were a total of 4892 proteins and 35,562 peptides (Supplementary Figure S1) identified from the AL-group. There were 4077 protein groups on average in the AL-λ group but 2250 protein groups on average in the control group. There were 2765 protein groups existing in both the control and AL-λ group, but 2127 protein groups only existed in AL-λ patents (Figure 1d). Patients can be diagnosed with different disease types according to statistical analysis results and separated from control patients (Figure 1e); the clustering results were consistent with the clinical diagnosis results. Four AL-λ patients expressed more proteins than the control group, which can distinguish control patients with AL patients easily with heat maps that drawing according to the protein abundance (Figure 1f). There was a big difference between the control and AL-λ groups in protein profiling. We can obviously distinguish control patients from AL patients with all identified proteins according to the primary proteomics results.

### 2.2. Subtype AL-λ Patients by Amyloid Fiber Protein

**Figure 2:**
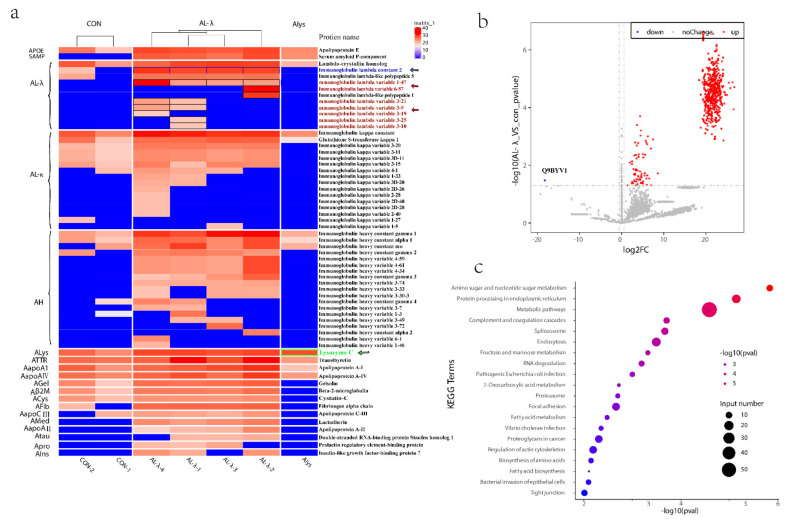
(**a**). Heat map of amyloid fiber proteins identified in control patients and renal amyloidosis patients, including Alys patient, Ig λ light-chain variable and Ig λ light-chain constant region fragments, shown in black border. (**b**). Volcano Plot of differentially expressed proteins with 2 control and 4 AL-λ patients. Gray dotted lines show *p* value < 0.05 and 1.5-fold change cut-offs. Up-regulated proteins are in red while the only down-regulated protein (Q9BYV1) is in blue. (**c**). Top 20 pathways highlighting the differentially expressed protein pathways between 2 control and 4 AL-λ patients.

We further analyzed the amyloid fiber proteins expressed in the control and AL-λ amyloidosis patients. Even though there was only one case of Alys patient, because it is so rare, we put it into analysis together. Apolipoprotein E protein and serum amyloid P component (SAP) were present in all four cases. Most amyloid fiber proteins detected in this study were Ig light-chain variable region fragments in four AL-λ patients. There was one Ig λ light-chain constant region fragments present in all four patients (Figure 2a, blue arrow). Ig λ light-chain variable 1-47 (IGLV 1-47) (Figure 2a, red arrow) was present in all four cases but Ig λ light-chain variable 3-9 (IGLV 3-9) (Figure 2a, red arrow) and Ig λ light-chain variable 3-21(IGLV 3-21) (Figure 2a, red arrow) were only present in the AL-λ-1/AL-λ-4 patient. Ig λ light-chain variable 3-10 (IGLV 3-10) (Figure 2a, red arrow) and Ig λ light-chain variable 3-24 (IGLV 3-24) (Figure 2a, red arrow) was only present in the AL-λ-1 patient (Figure 2a). Lysosome was only expressed compared with other amyloid fibers in the Alys patient (Figure 2a, green arrow). Here, we could find Ig κ light-chains C region and κ light-chain V region in the AL-λ patient but κ light-chain was not expressed significantly compared with λ light-chain (Figure 2a). Most of the antibodies (antibody-based methods) used in clinical diagnosis were from the Ig light-chain constant region; these light-chain variable fragments could not be detected by IF or IHC method, whereas we could find the light-chain fragments in different cases clearly. We can see the amyloid fiber proteins identified in all the patients from Figure 2a. The amyloid fiber proteins were expressed obviously differently, even though only in the same AL type patients, and we can accurately subtype amyloidosis patients into molecular types.

We further analyzed all the proteins differently expressed between control and Al-λ patients. It is very interesting that there was only one down-regulated protein significantly expressed in the AL-λ groups: Alanine-Glyoxylate Aminotransferase 2 (AGTX2) (Figure 2b).

### 2.3. Enrichment Analysis of Pathogeny

AL amyloidosis is a metabolic disease; although it is associated with a single clone of proliferating plasma cells, the majority of patients do not develop a malignant disease as multiple myeloma but suffer from monoclonal gammopathy of undetermined significance (MGUS). This observation indicates that some additional factors are required for the development of amyloid deposits. One such factor seems to be the ability of LCs to be bound by macrophages; where intact LCs were metabolized into insoluble and unstable fragments with biochemical properties that allow them to form amyloid fibrils, λ LCs were more amyloidogenic than the κ ones [4]. In order to further explore the pathogenesis of renal amyloidosis, GO and KEGG pathway enrichment analysis were performed on the differential proteins expressed in control patients and AL-λ patients.

GO analysis showed that the most enriched molecular functions were RNA binding, cytoskeleton binding, cell adhesion molecule binding, and cadherin protein binding and other functions, as shown in Supplementary Figure S2a; the most enriched biological processes were: establishment of localization in cell, vesicle-mediated transport, metabolic process, small molecule metabolic process, establishment of localization, cellular metabolic process, cellular process, regulated exocytosis, transport, localization, exocytosis, neutrophil degranulation, neutrophil activation involved in immune response, organic substance metabolic process and myeloid cell activation involved in immune response, as shown in Supplementary Figure S2b. These biological processes were closely related with amyloidosis.

AL-λ patients often had multiple myeloma or plasma cell malignant hyperplasia, which was a kind of primary amyloidosis. Immunoglobulin-related amyloidosis was related to plasma cell diseases, and the primary treatment was to control plasmacytosis. The pathways enriched by KEGG analysis were mainly metabolic pathways, proteasome, spliceosome, focal adhesion, biosynthesis of amino acids, amoebiasis, ECM-receptor interaction, lysosome, arginine and proline metabolism, complement and coagulation cascades, Parkinson’s disease, prion diseases, endocytosis, amino sugar and nucleotide sugar metabolism, pentose phosphate pathway, protein processing in endoplasmic reticulum, etc. (Figure 2c). These enriched pathways were closely related to the development of AL amyloidosis and its pathogenesis matched.

### 2.4. Mass Spectrometry Typing Lysozyme Amyloidosis

**Figure 3:**
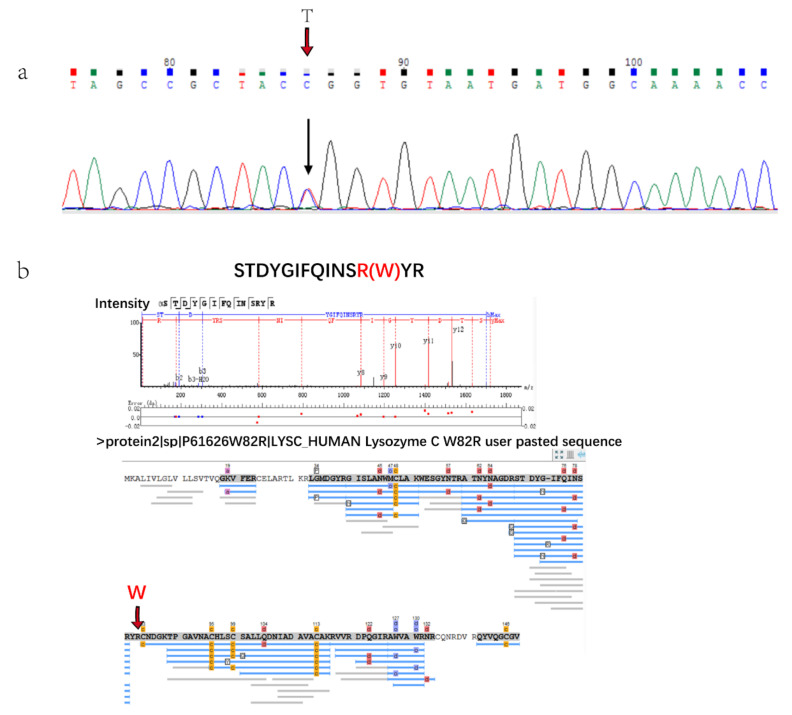
(**a**). Gene sequencing results of Alys patient base transitions from T to C. (**b**). Results of protein database searching by Peaks software. Peptide of p.Trp82Arg mutation was identified.

There was a patient with hereditary lysozyme amyloidosis in this study who was diagnosed and typed by genetic analysis, because IHC cannot type without antibody. The gene sequencing results showed that there was a single base transitions from T to C at the first position of codon 82 (TGG/CGG) of exon 2 (Figure 3a). The amyloid fiber protein analysis showed that amyloid precursors were clearly distinguished from the control group and AL patients. Lysozyme C was the only highly expressed amyloid fiber protein in Alys patients (Figure 2a) and it was not expressed or expressed very low compared with other amyloid fiber protein in other patients. The protein expressed by the p.Trp 82Arg mutation could be detected by mass spectrometry (Figure 3b) in Chinese Lysozyme amyloidosis patients for the first time. These results verified the clinical diagnosis.

## 3. Discussion

The number of papers using FFPE tissues in proteomic analysis has been growing in recent years. The interest to apply proteomic analysis to FFPE tissues lies in the easy accessibility of a great number of samples from archives [24]. Renal amyloidosis FFPE tissues are trace clinical samples (15–30 glomeruli) and Alys is an extremely rare type amyloidosis, so there are only about five glomeruli for further study. The micro-sample pretreatment technology is important for proteomics analysis. The conventional sample pretreatment process has many steps, and low amounts of samples can be easily lost during the preparation steps. As a result, micro-sample pretreatment technology has been optimized and improved on the basis of conventional techniques: a. Heptane was used instead of xylene to reduce toxicity, and the volume ratio of heptane and methanol was adjusted (500 μL n-heptane was adjusted to 1000 μL) (the conventional ratio was 10:1, adjusted to 5:1) to remove paraffin fully avoiding interference with mass spectrometry analysis; b. The sample-transfer times was reduced, and the pre-treatment process was carried out all in one centrifuge tube to minimize sample loss as well as avoiding contamination; c. Alkaline medium was used to facilitate protein crosslinking reversion, and the heat temperatures was set higher than 80 °C for 2 h. Clinical micro-samples can be analyzed by proteomics with the pre-treatment method [4,24,25].

Renal amyloidosis is a rare disease and we obtained very limited samples. Even with such small sample size, we still obtained very meaningful data from mass spectrometer results. The total protein expressed level from proteomics results between control and AL patients can type amyloidosis patients. The number of proteins identified for control samples is lower than the number of proteins identified for the AL samples, and there is a significant difference between the two groups in the number of identified proteins. The glomeruli samples of AL patients were completely different from patients with minor glomerular disease, as shown in (Supplementary Figure S3). There was large amount of amyloid fiber precipitation in the glomerulus of AL amyloidosis patients, which led to great pathological changes in the glomerulus; however, there was not any amyloid fiber in the glomerulus of control patients. AL amyloidosis is typically found in individuals with monoclonal gammopathy, a disorder that is characterized by the proliferation of clonal plasma cells. It was resulted in the increased production of clonal immunoglobulin light chains; these light chains aggregate into amyloid fibrils, leading to organ damage. This process is complex, and it can be influenced by several factors, such as mutations that destabilize the native protein structure and expose hydrophobic and protease-sensitive regions, increasing protein concentrations, owing to either greater protein synthesis or reduced clearance [26,27]. When intracellular proteostasis and/or extracellular proteostasis fail, protein aggregation might occur. On one hand, we hypothesized that the deposition of amyloid in the kidney stimulated the injury of endothelial cells, which then activated the complement system, and stimulated the release of a large numbers of inflammatory factors. On the other hand, the body needs to remove and degrade excess amyloid. Amyloid deposits are in general persistent and unusually resistant to degradation. However, slow natural clearance of amyloid deposits, by endogenous immunological mechanisms in which macrophages play an important part, does occur. In these processes of pathological change in AL-λ patients, many biological pathways participate and it produces much more proteins compared with control patients. We can also find the related pathways from KEGG analysis (Figure 2c) which are related to the greater protein synthesis or reduced clearance process. Hence, the great pathological change of amyloid fibers leads to the difference in the numbers of protein identification between control and AL patients.

Immunoglobulin (Ig) light chain (LC) amyloidosis (AL) is characterized by an abnormal extracellular deposition of a monoclonal Ig LC protein of either lambda (λ) or kappa (κ) isotype [8]. According to the results of FFPE-based proteomics retrospective diagnosis analysis, it can distinguish AL patients from control patients. Criteria for diagnosis and typing of amyloidosis was AL λ light-chain amyloid which contains large spectra of Ig lambda light-chain C region with or without λ light-chain V region, and absence of significant κ light-chains [19]. Four AL-λ patients expressed high abundance λ subtype proteins. The deposited LC variable region (LCV) is clonotypic and unique to each patient, and thought to be the primary pathogenic driver of the disease [28]. Maybe it can explain the indolent/localized nature of this disease with MS results. FFPE-based proteomics can not only distinguish patients with minor glomerular disease from AL patients but also can show other amyloid fiber proteins in every AL patient for further molecular subtypes. Here we also found Ig heavy chain in four AL-λ patients’ proteomic results. Heavy chain amyloidosis (AH amyloidosis) caused by renal deposition of a monoclonal immunoglobulin heavy chain, is a much rarer type of immunoglobulin-related amyloidosis, with only a limited number of cases having been reported [29]. Criteria for diagnosis and typing AH: heavy chain: λ, a, µ, with large number of spectra, in comparison with light-chain spectra [19]. Four AL-λ cases were typed AL-λ instead of AH or mixed AH+AL amyloidosis because staining for immunoglobulin light chain is the first step—if renal immunoglobulin light chain staining was positive, it would be typed AL amyloidosis. We can find Ig heavy chain fragments form proteomics results, and FFPE-based proteomics was useful to reach a final diagnosis and helpful to explain the mechanism why heavy chain staining gave the negative results of IHC. From the results of FFPE-proteomics, there were other amyloid precursors but low abundance compared with Ig λ light-chain. This result suggests that one type of renal amyloidosis may contain one or several pathogenic amyloid fiber proteins. It would be difficult to type the patient by immunofluorescence or immunohistochemistry when there were two or more pathogenic amyloid proteins. The application of proteomics can perform a more comprehensive and in-depth analysis of all amyloid precursor proteins of patients without limited in antibodies. Compared with the control group patients, the profile of amyloid precursor proteins detected by proteomics was different in four AL-λ cases. Although four patients were the same disease type, the amyloidosis protein types and abundances were not exactly the same; in addition, the abundance of Ig λ light-chain detected in the four patients were different, too. The AL-λ type cases can be further analyzed according to different λ amyloid precursor proteins to provide accurate clinical classification and individualized treatment, and to further explore its pathogenesis.

AGXT2 was the only down-regulated protein in differentially expressed proteins and was mainly expressed in the kidney. It was reported that AGXT2 may play a part in the progression of renal diseases through affecting ADMA (Asymmetric dimethylarginine)/SDMA (symmetric dimethylarginine) level [30]. AL amyloidosis results from extra-cellular deposition of fibril-forming monoclonal Ig LC, usually produced by a small plasma cell clone [16]. The role of AGXT2 in renal function is worthy of further investigations especially in AL amyloidosis, as maybe it had something to do with the occurrence and development of the AL amyloidosis. Here we provide an experimental basis for further research.

Lysozyme amyloidosis (Alys) is a systemic amyloidosis, and one of the rarest types of such. Alys is regarded as a type of autosomal dominant genetic disease caused by mutations in genes encoding proteins [25]. Alys is a type of hereditary amyloidosis that is extremely rare in clinical practice, first described in 1993 by Pepys et al. [31]. It is so rare that only about thirty families have been noted across the world [10]. The hereditary amyloidosis was usually caused by genetic mutations that lead to amino acid mutations in the encoded protein, which affect the three-dimensional structure of the protein and its interaction with other proteins, forming amyloid deposits [19,22]. The diagnosis and treatment of Alys are significantly different from other types, which directly affect the prognosis. Liver or kidney transplantation may be useful as a palliative method for patients with spontaneous liver rupture or renal failure [10]. Proteomics can directly detect pathogenic amyloid fibrin precursors and accurately type hereditary amyloidosis, while traditional IHC technology often cannot type for lack of hereditary amyloidosis antibody. In this study, the patient was finally typed by gene sequencing technology because of lack of antibody for IHC. Whether genetic abnormalities will be translated into protein level is difficult to predict. Here we have identified the p.Trp 82Arg variant genetic mutation peptide by the mass spectrometry for the first time in a Chinese patient. FFPE-based proteomics is particularly advantageous in the diagnosis and type of hereditary amyloidosis. Proteomics is supplementary to immunohistochemistry and gene sequencing for amyloid typing, and it can achieve individualized and accurate diagnosis of amyloidosis. Since this study was a small sample size experiment, we successfully applied proteomics for disease diagnosis and molecular typing. Subsequently, we need to conduct large-scale clinical samples for verification.

## 4. Materials and Methods

### 4.1. Reagents and Chemicals

Ammonium bicarbonate and acetonitrile (ACN) were purchased from Fluka (St. Louis, MO, USA). Trypsin and dithiothreitol (DTT) were purchased from Promega (Madison, WI, USA). Iodoacetamide (IAA), trifluoroacetic acid (TFA) and heptane were purchased from Sigma (St. Louis, MO, USA).

### 4.2. FFPE Tissue Collection

FFPE renal biopsies of five cases were clinically diagnosed renal amyloidosis tissue samples (including 4 cases of AL-λ type and 1 case of hereditary ALys amyloidosis); two FFPE tissues with minor glomerular disease were used as control samples. There were several glomeruli in every FFPE slide which were heterogeneity between samples of different patients. The research study was implemented in accordance with the relevant guidelines and regulations. All FFPE samples were provided by Ruijin Hospital affiliated with Shanghai Jiao Tong University School of Medicine. The utilization of anonymized archival material in retrospective studies was approved by the Ethics Committee of Ruijin Hospital affiliated with Shanghai Jiao Tong University School of Medicine.

### 4.3. Sample Preparation for Proteomic Analysis

The method was conducted as follows: Collect the FFPE pathological section into a 1.5 mL centrifuge tube, add 1000 μL heptane, vortex vigorously and let stand at room temperature for 1 h. Add 100 μL methanol, centrifuge vigorously and discard the supernatant. Repeat this step 2–3 times and transfer to the paraffin. After being completely taken off, blow-dry the tissue in a fume hood. Add 100 μL EXB (QIAGEN) extraction solution (beta-mercaptoethanol final concentration 4%), incubate on ice for 5 min and vortex. Incubate the thermomixer at 100 °C for 30 min, and then continue to incubate at 80 °C at 750 rpm for 2.5 h. After that, centrifuge at 14,000× *g*, 4 °C for 15 min, transfer the supernatant to a new 1.5 mL centrifuge tube, add 1 μL 1 M DTT (in 100 mM NH_4_HCO_3_) and incubate at 37 °C, rotating for an hour at a speed of 750 rpm. Add 1 μL 500 mM IAA (in 100 mM NH_4_HCO_3_) and react in the dark for 45 min at room temperature. After the reaction is over, add 600 μL of acetone, vortex vigorously for 10 s and place in a refrigerator at −20 °C overnight. Centrifuge at 14,000× *g* for 30 min the next day, and discard the supernatant; wash three times, discard the supernatant and finally blow-dry the protein pellet. Resuspend the protein with 20 μL, 100 mM NH_4_HCO_3_, and add 2 μL trypsin (0.25 μg/μL). Incubate overnight at 37 °C (16–18 h). Add 2 μL of 10% (*v/v*) TFA afterwards. Desalt using Zip tip C18.

### 4.4. Mass Spectrometry Conditions and Methods

The eluted peptides were lyophilized using a SpeedVac (Thermo Savant) and resuspended in 10 μL of 1% formic acid/5% acetonitrile. All mass spectrometric experiments were performed on a Thermo Fusion Lumos mass spectrometer connected to an Easy-nLC 1200 via an Easy Spray (Thermo Fisher Scientific). The peptides mixture was loaded onto a 15 cm column with 0.075 mm inner diameter column packed with C18 2-μm reversed phase resins (PepMap RSLC), and separated within a 60 min linear gradient from 95% solvent A (0.1% formic acid/2% acetonitrile/98% water) to 28% solvent B (0.1% formic acid/80% acetonitrile) at a flow rate of 300 nL/min. The spray voltage was set to 2.1 KV and the temperature of ion transfer capillary was 275 °C, and RF lens was 60%. The mass spectrometer was operated in positive ion mode and employed in the data-dependent mode to automatically switch between MS and MS/MS using the Tune and Xcalibur 4.0.27.19 software package. One full MS scan from 350 to 1500 m/z was acquired at high resolution R = 60,000 (defined at m/z = 400), followed by fragmentation of the twenty most abundant multiply charged ions (singly charged ions and ions with unassigned charge states were excluded), for ions with charge states 2–7 and collision energy of 30%. Dynamic exclusion was used automatically.

### 4.5. Database Search and Data Analysis

All MS/MS ion spectra were analyzed using Peaks Studio 8.0 software (Bioinformatics Solutions Inc., Waterloo, ON, Canada) for processing, de novo sequencing and database searching. Resulting sequences were searched against the UniProt Human Proteome database (downloaded 5 May 2018) with mass error tolerances of 10 ppm and 0.02 Da for parent and fragment, respectively. The digestion enzyme trypsin allowed for two missed tryptic cleavages, Carbamidomethyl of cysteine specified as a fixed modification, and Oxidation of methionine and acetyl of the N-terminus as variable modifications. FDR estimation was enabled. Peptides were filtered for −10log *p* ≥ 20, and proteins were filtered for −10log *p* ≥ 15 and one unique peptide. For all experiments, this gave an FDR of <1% at the peptide-spectrum match level. Proteins sharing significant peptide evidence were grouped into clusters.

### 4.6. Statistical Analysis and Enrichment Analysis

Screening for differentially expressed proteins uses 1.5-times fold change as the criterion for selecting differentially expressed proteins. When the fold change (protein abundance ratio) ≥ 1.5 and *p* < 0.05, it is defined as up-regulation of protein. Principal component analysis was performed on the relative expression of the protein in the samples of the control group and the disease group, and clustering heat maps were drawn for all the identified proteins and proteins related to renal amyloidosis.

Gene Ontology (GO) and Kyoto Encyclopedia of Genes and Genomes (KEGG) pathway enrichment analysis were applied to significantly differentially expressed proteins using Fisher’s exact test in R programming language. Enriched GO terms and KEGG pathways are filtered out using *p*-value < 0.05.

### 4.7. Gene Sequences Analysis

The gene sequences were determined by the Illumina Hiseq 3000 sequencer using Roche Company’s proprietary custom-made Nimblegen targeted capture probe in the gene sequencing. BWA software, GATK 3.1.1 mutation detection software and ANNOVAR software were used to compare the reads of the sequencing results to the known human reference genome sequence hg19 (UCSC), annotate the mutation sites and align the positions. Points are graded for variation, respectively. According to site annotation information combined with biology, genetics and clinical characterization information, suspicious mutation sites were comprehensively analyzed and screened. The selected suspicious sites were verified using ABI 3730 sequencer for Sanger sequencing.

## 5. Conclusions

This retrospective pilot study demonstrates that FFPE-based proteomics and statistics analysis method for trace renal amyloidosis is reliable and specific. However, this method has some limitations—the sample size is small and the instrument is expensive, which requires professional operation and analysis. With significant technical advantages over immunohistochemistry, it has great prospects in clinical application. Even though the method cannot be applied to clinical practice on a large scale, it is a step in the right direction.

## Data Availability

The data sets generated and/or analyzed in the current study are available on request from the corresponding author.

## Supplementary Materials

The following are available online, Figure S1: Identified protein in control and AL-λ groups. Figure S2: (a) Go enrichment analysis bubble plot for molecular functions. (b) Go enrichment analysis bubble plot for biological processes. Figure S3: Light micrograph and Immunofluorescent staining for lambda light chain in kidney biopsy.

## Sample Availability

Not available.
