# Supplementary material for: A Pilot Study of Rare Renal Amyloidosis Based on FFPE Proteomics
Source: Molecules. 2021 Nov 29;26(23):7234. doi: 10.3390/molecules26237234 (PMC8659071; doi:10.3390/molecules26237234)
Supplement: Supplementary file 1 [file molecules-26-07234-s001.zip › molecules-1415528-supplementary.pdf]

molecules

Supplementary Materials

A Pilot Study of Rare Renal Amyloidosis Based on FFPE Proteomics

Shuang Meng [1,†], Wenwen Xia [2,†], Li Xia [1], Li Zhou [1], Jing Xu [3], Xiaoxia Pan [3,*] and Liyuan Meng [1,*]

[1] Department of Core Facility of Basic Medical Sciences, Shanghai Jiao Tong University School of Basic
Medicine, Shanghai 200025, China; mengshuang92@shsmu.edu.cn (S.M.); lixia@shsmu.edu.cn (L.X.); zhouli_bit@shsmu.edu.cn (L.Z.)
[2] Department of Pathology, Ninth People's Hospital, Shanghai Jiao Tong University School of Medicine, Shanghai 200011, China; xiawenwenliuyedao@163.com
[3] Department of Nephrology, Ruijin Hospital, Shanghai Jiao Tong University School of Medicine, Shanghai 200025, China; xj11564@rjh.com.cn
* Correspondence: pxx10768@rjh.com.cn (X.P.); mengliyuan@shsmu.edu.cn (L.M.); Tel.: +86-21-64370045 (X.P.); +86-21-63846590 (L.M.)
† These authors contributed equally to this work.

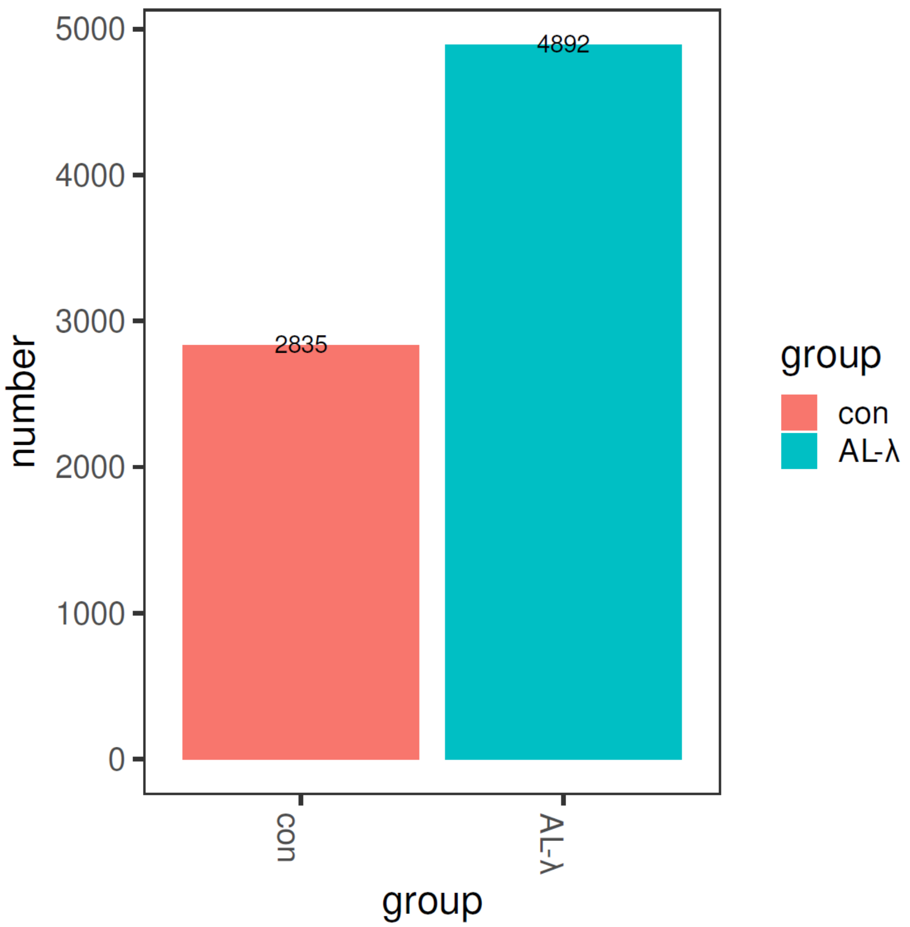

Figure S1. Identified protein in control and AL-λ groups.

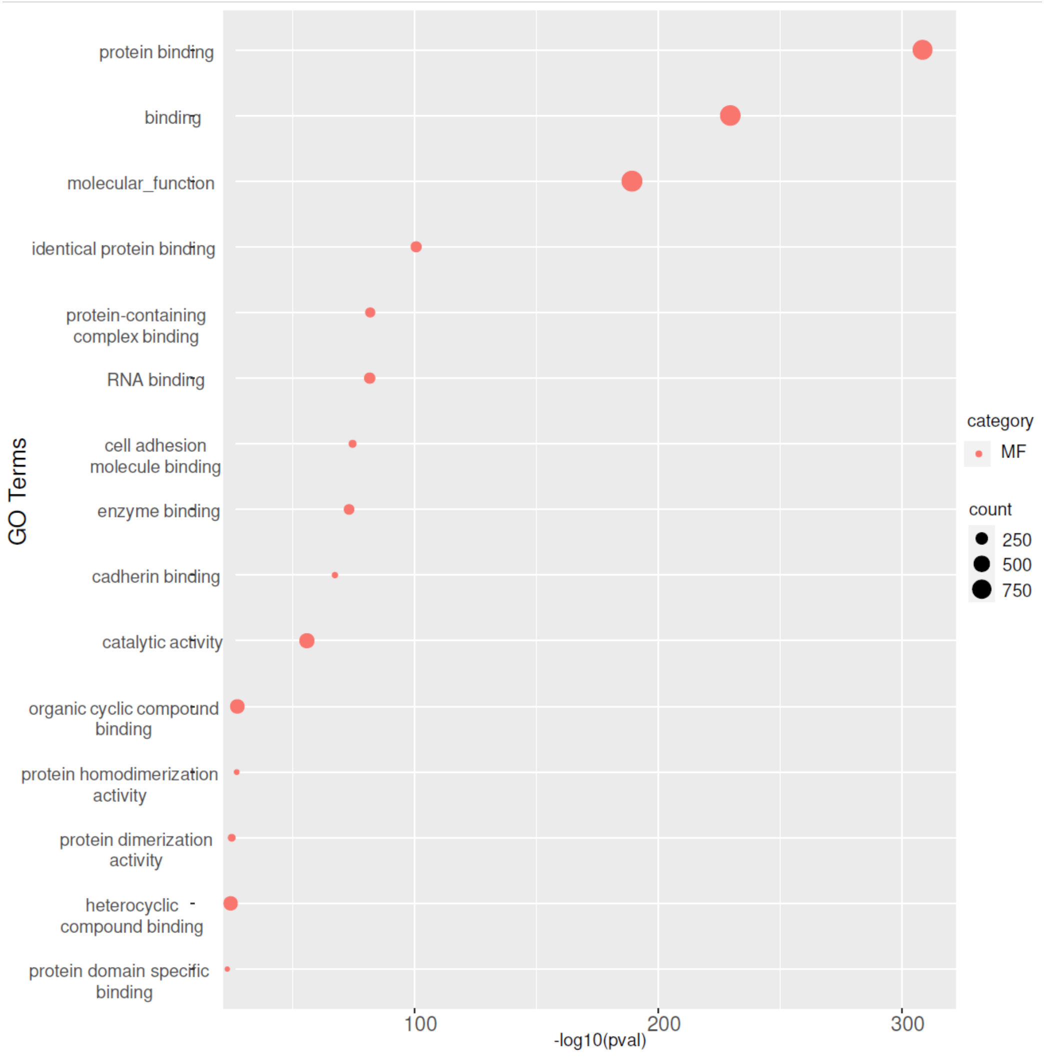
GO Terms
protein binding
binding
molecular_function
identical protein binding
protein-containing complex binding
RNA binding
cell adhesion molecule binding
enzyme binding
cadherin binding
catalytic activity
organic cyclic compound binding
protein homodimerization activity
protein dimerization activity
heterocyclic compound binding
protein domain specific binding
100
200
300
-log10(pval)
category
MF
count
250
500
750

(a)

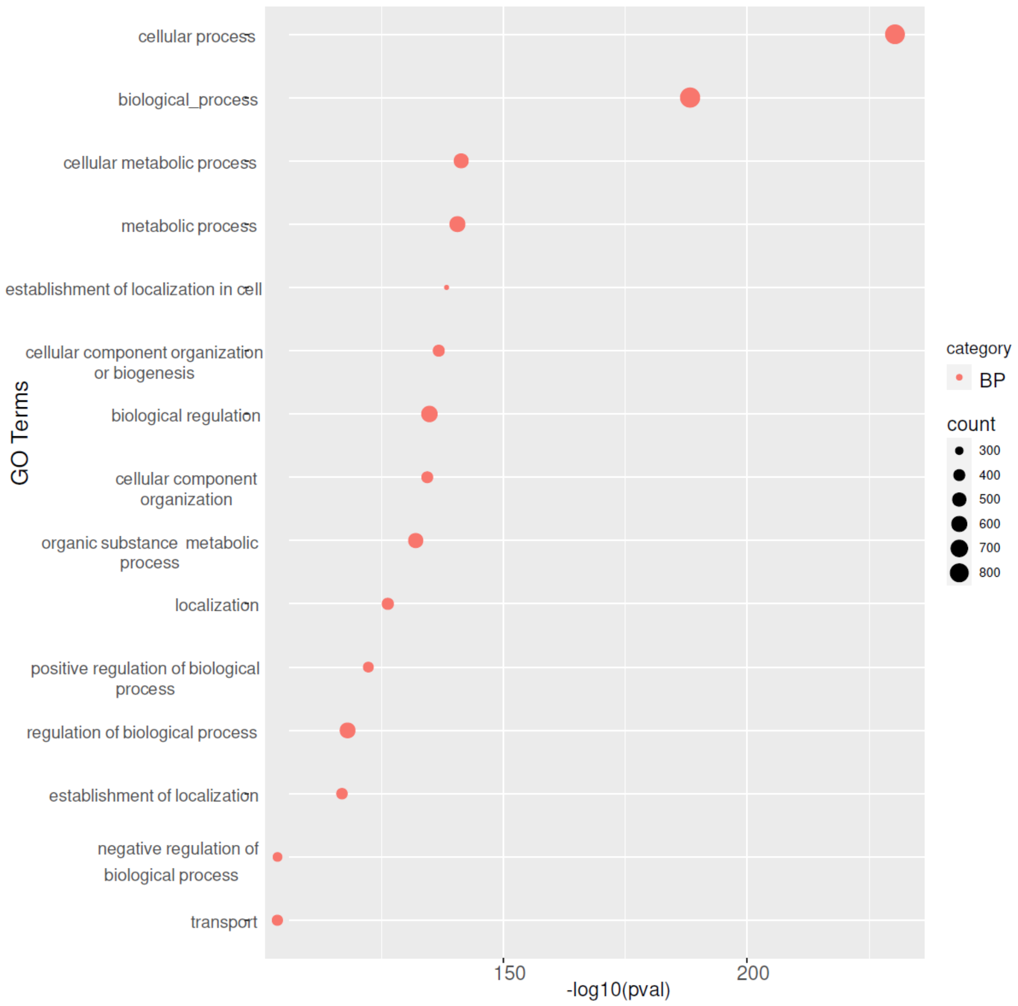

(b)

Figure S2. (a) . Go enrichment analysis bubble plot for molecular functions. **(b)** . Go enrichment analysis bubble plot for biological processes.

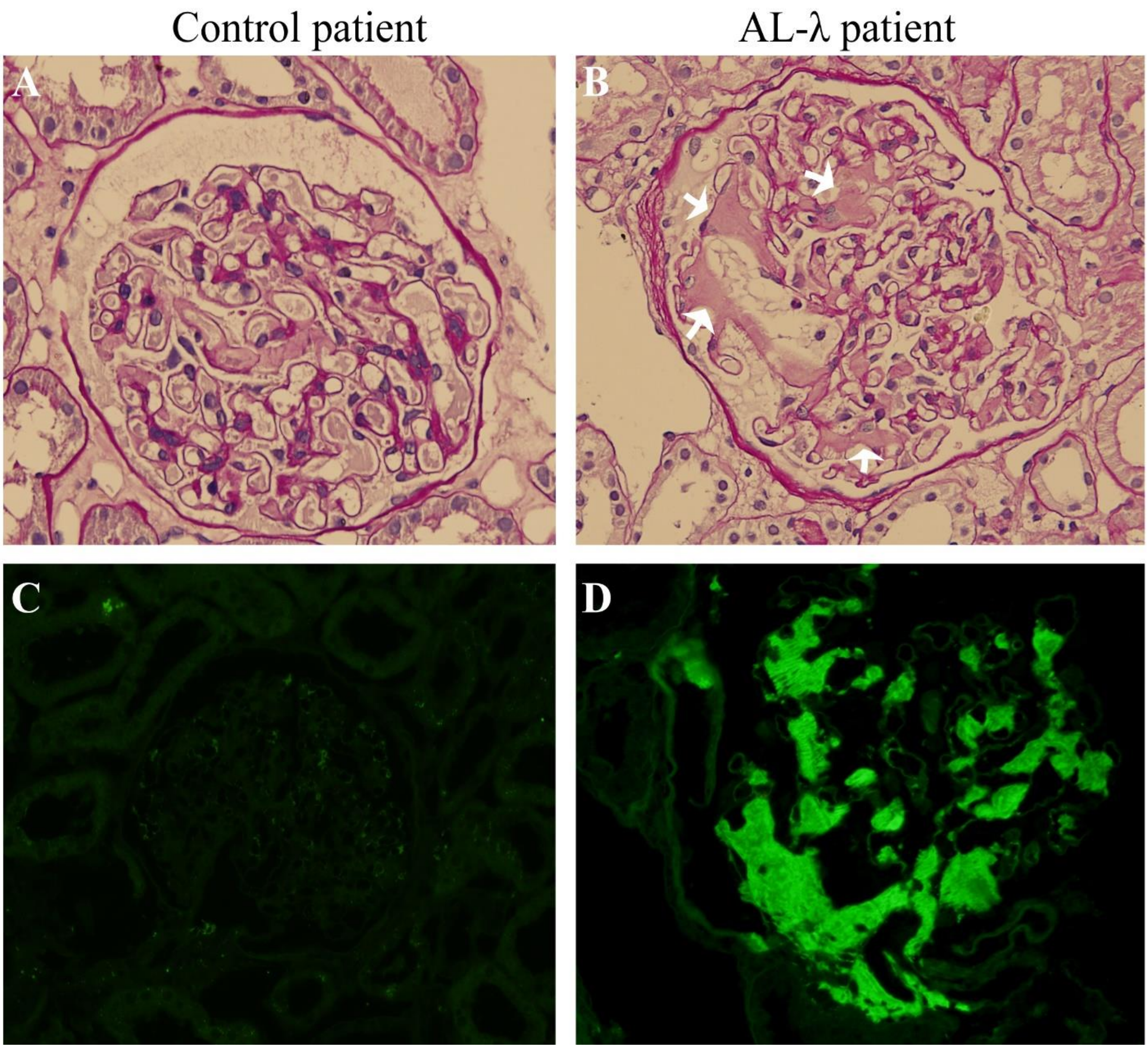

Figure S3: Light micrograph and Immunofluorescent staining for lambda light chain in kidney biopsy. a. light micrograph of glomerulus from a patient with minor glomerular disease showing no glomerular changes; b. mesangial and arteriolar amyloid deposition in patient with amyloidosis (white arrow); c. no lambda light chain deposition in patient with minor glomerular disease; d. positive mesangial and arteriolar lambda light chain deposition in patient with amyloidosis. (a. b. PAS 400´; c. d. light chain lambda 400´)
